# Genomic Features of Interstitial Deletions of Chromosome 9q in Acute Myeloid Leukemia

**DOI:** 10.1159/000525010

**Published:** 2022-06-08

**Authors:** Zhongxia Qi, Kwun Wah Wen, Anita Ki, Sonam Prakash, Scott Kogan, Jingwei Yu

**Affiliations:** ^a^Department of Laboratory Medicine, University of California San Francisco, San Francisco, California, USA; ^b^Department of Pathology, University of California San Francisco, San Francisco, California, USA; ^c^Clinical Cytogenetics Laboratory, University of California San Francisco Medical Center, San Francisco, California, USA

**Keywords:** Acute myeloid leukemia, Deletion of 9q, Genomic analyses, Common overlapped deletion region, Cancer-related genes

## Abstract

Interstitial deletion in the long arm of chromosome 9 [del(9q)] is a fairly common cytogenetic finding associated with acute myeloid leukemia (AML), seen in approximately 2–5% of AML patients. However, the genomic features of the deletion remain largely unknown. Using chromosome analysis, single nucleotide polymorphism microarray, and next-generation sequencing, we characterized del(9q)s and other genomic alterations in 9 AML patients. We found several distinct features of the del(9q)s. The proximal breakpoints of the deletions are clustered within a 2.5-Mb region (chr9: 68,513,625–70,984,372; GRCh37) enriched with segmental duplications, which may represent a “hotspot” for genomic rearrangements. However, the distal breakpoints of the deletions vary significantly. In addition, the overall deleted region could be divided into a 14.4-Mb proximal constitutional region (chr9: 70,950,015–85,397,699; 9q21.11q21.32) and a 24.0-Mb distal oncogenic region (chr9: 85,397,700–109,427,261; 9q21.32q31.1). We further identified a 6.8-Mb common overlapped deletion region (CODR) in the distal region (chr9: 90,590,650–97,366,400). This CODR carries multiple genes that are reportedly involved in cancer pathogenesis. The prognostic value of the del(9q) in AML apparently depends on additional genomic alterations in the patients.

## Introduction

Interstitial deletion in the long arm of chromosome 9 [del(9q)] is a recurrent finding in acute myeloid leukemia (AML). The deletion has been reported to occur predominantly in myeloid disorders, frequently in AMLs with t(8;21) translocation. It can occur solely or with other abnormalities as a primary or secondary event. However, the pathogenic role of del(9q) is unclear despite numerous studies of the deletion [Mecucci et al., 1984; Akashi et al., 1992; Kwong et al., 1993; Peniket et al., 2005; Balk et al., 2019]. In addition, the genomic features of the deletion remain mostly undefined, hindering further understanding of the pathogenic significance of the deletion. Integrated genomic analyses, including chromosome analysis, single-nucleotide polymorphism microarray (SNP array), and next-generation sequencing (NGS), make it possible to delineate genomic abnormalities in detail. Using these technologies, we characterized del(9q)s along with other genomic alterations in 9 patients with AML. Our studies revealed distinct genomic features of the deletions, which have not been described previously. These features include clustered proximal and variable distal breakpoints, proximal constitutional and distal oncogenic regions, and a common overlapped deletion region (CODR) that carries known cancer-related genes. In addition, we also detected various additional genomic alterations in these patients, which may affect the prognostic value of the del(9q).

## Materials and Methods

### Patients

Nine patients with AML and del(9q) from clinical cases tested at the University of California San Francisco (UCSF) Cytogenetics Laboratory between 2012 and 2021 were selected for this study (Table 1).

### Chromosome Analysis

Chromosome analysis of bone marrows was performed following standard cytogenetic methods. Chromosome abnormalities were described according to the International System for Human Cytogenomic Nomenclature [ISCN, 2020].

### DNA Extraction and SNP Array

Genomic DNA was extracted from bone marrows using Qiagen EZ1 DNA kit. SNP array was performed and analyzed based on human genome build GRCh37/hg19 using Illumina CytoSNP-850K BeadChip and the package analytical software (BlueFuse Multi) following the manufacturer's instruction.

### NGS Pathogenic Variant Analysis

AML NGS gene panel studies were clinically requested for all 9 patients, and the tests were performed in UCSF Clinical Cancer Genomic Laboratory, ARUP Laboratories or Foundation Medicine. The gene panels included *ANKRD26, ASXL1, ASXL2, BCOR, BCORL1, BRAF, CALR, CBL, CBLB, CEBPA, CSF3R, CUX1, DDX41, DNMT1, DNMT3A, ELANE, ETNK1, ETV6, EZH2, FBXW7, FLT3, GATA1, GATA2, GNAS, HNRNPK, IDH1, IDH2, IL7R, JAK1, JAK2, JAK3, KDM6A, KIT, KMT2A, KRAS, LUC7L2, MPL, NOTCH1, NPM1, NRAS, NSD1, PHF6, PIGA, PRPF40B, PRPF8, PTPN11, RAD21, RUNX1, SETBP1, SF3B1, SH2B3, SMC1A, SMC3, SRSF2, STAG2, STAT3, STAT5B, SUZ12, TET2, TP53, U2AF1, U2AF2, WT1,* and *ZRSR2*.

## Results

**Fig. 1:**
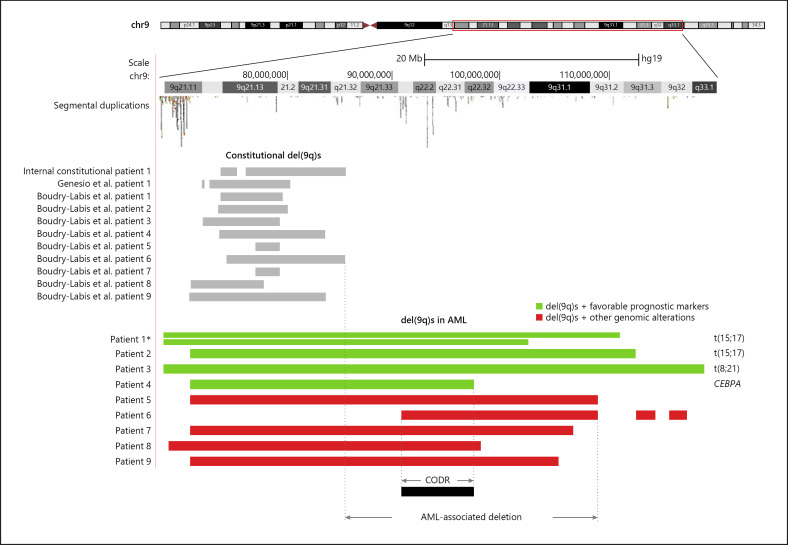
Genomic features of del(9q). The distribution of segmental duplications is based on the Segmental Duplication Track in UCSC genome browser (https://genome.ucsc.edu). *, Patient 1 carried 2 different 9q deletions in 2 separate clones. CODR, common overlapped deletion region.

Interstitial 9q deletions were detected by chromosome analysis in all 9 AML patients. The deletions were confirmed and their sizes and breakpoints were defined by SNP array (Table 1). All deletions were located between 9q13 and 9q33.1, ranging from 18.2 to 50.2 Mb in size. However, the proximal and distal breakpoints of the deletions apparently showed different genomic features. The vast majority of the proximal breakpoints were clustered within a 2.5-Mb region (chr9: 68,513,625–70,984,372) which is enriched with segmental duplications; whereas, the distal breakpoints varied significantly (Fig. 1).

By reviewing literature and our internal constitutional microarray database, we found multiple constitutional interstitial del(9q)s that were located within an approximately 14.4-Mb region between 9q21.11 and 9q21.32 (chr9: 70,950,015–85,397,699) [Boudry-Labis et al., 2013; Genesio et al., 2015]. These constitutional deletions completely overlap with the proximal region of the AML-associated del(9q)s in this study (Fig. 1). However, no neoplastic phenotypes were reported in the individuals with the constitutional deletions, suggesting that this proximal deletion region may not play a pathogenic role in AML. Excluding the proximal constitutional deletion region, the potential oncogenic part of the del(9q)s could be narrowed down to the remaining 24.0-Mb distal deletion region (chr9:85,397,700–109,427,261) within 9q21.32q31.1 (Fig. 1), consistent with a previously reported common deleted region of del(9q)s in AML [Peniket et al., 2005]. In addition, we identified a 6.8-Mb CODR (chr9: 90,590,650–97,366,400) within the distal deletions in all 9 patients. The CODR contains 118 known genes, including cancer-related genes *GADD45G, WNK2, MIRLET7A1, MIRLET7D*, and *MIRLET7F1* (https://dosage.clinicalgenome.org). Loss of function or reduction of expression of these 5 genes have been reported to be associated with cancer pathogenesis (see Discussion).

In addition, all 9 patients in this study also carried additional genomic alterations, including chromosomal abnormalities, genomic copy number alterations, copy number neutral loss of heterozygosities, and gene sequence pathogenic variants (Table 1). The occurring order of the additional abnormalities in relation to del(9q) could not be determined in most of the patients, except for patient 2, in which del(9q) was a secondary genomic alteration based on chromosomal findings (Table 1).

## Discussion

The 9q deletions in AML apparently carry unique genomic features that have not been reported. The proximal breakpoints of the deletions are clustered in an approximately 2.5-Mb region which is enriched with short segmental duplications (Fig. 1). Such condensed segmental duplications may facilitate nonallelic homologous recombination, resulting in increased risk for del(9q)s with relatively uniform proximal breakpoints. This region may represent a “hotspot” for genomic rearrangements in the human genome. However, the distal breakpoints of the deletions may occur at various loci through different mechanisms. Interestingly, patient 1 of this study carried 2 different del(9q)s with the same proximal breakpoint but different distal breakpoints (Fig. 1). The deletions could result from a proximal chromosome breakage followed by different distal breakages on separate sister chromatids during mitosis.

We identified a proximal deletion region in the AML-related 9q deletions, which overlaps with reported constitutional del(9q)s. The common phenotypic features associated with this deletion region include mental retardation with speech delay, epilepsy, autistic behavior, and moderate facial dysmorphology [Boudry-Labis et al., 2013]. However, the AML patients apparently did not show such constitutional phenotypic abnormalities. This could be explained by the fact that the AML-associated deletions would only occur in hematopoietic cells, and other cells/tissues of the AML patients were unaffected. Thus, these patients would not show the constitutional phenotypic abnormalities.

It is hypothesized that haploinsufficiency of critical gene(s) within del(9q)s plays a role in AML pathogenesis. Herold et al. [2017] demonstrated a significant impact of haploinsufficiency of del(9q), but were unable to detect pathogenic variants in the genes within the del(9q) region by exome sequencing. Nonetheless, efforts have been made to search for a critical region within 9q deletions, which is responsible for the pathogenesis of AML. Sweetser et al. [2005] identified a 2.4-Mb minimal common deletion region in 43 AML cases with del(9q), but they did not identify recurrent pathogenic variants in all 13 genes within/adjacent to this region either. A similar region was also reported by Naarmann-de Vries et al. [2019]. Our study suggests that the overall region involved in the del(9q)s can be divided into a proximal constitutional region and a distal oncogenic region. The latter contains a 6.8-Mb CODR that is approximately 3.0 Mb distal to the previously reported minimal common deletion region [Sweetser et al., 2005; Naarmann-de Vries et al., 2019]. The CODR carries 5 reported cancer-related genes. Among these genes, *GADD45G* encodes a DNA damage inducible protein in p53 signaling pathway associated with cell cycle arrest. Defects in the GADD45G pathway appear to be involved in initiation and progression of malignancies [Tamura et al., 2012]. WNK2 has been identified as a tumor suppressor in hepatocellular carcinoma, glioma, and pancreatic ductal adenocarcinoma [Zhou et al., 2019]. *MIRLET7A1, MIRLET7D*, and *MIRLET7F1* are microRNAs that were reported to negatively regulate *HRAS, KRAS*, and *NRAS* oncogenes. Reduced expression of these microRNAs was associated with adverse prognosis independent of disease stage in lung cancer [Takamizawa et al., 2004]. Further investigation of these and other genes involved in del(9q)s may lead to a better understanding of the pathogenesis and progression of AML with del(9q)s.

Sole del(9q) seen in AML is believed to be an early event in leukemogenesis and associated with an intermediate risk [Peniket et al., 2005; Herold et al., 2017]. However, additional genomic alterations frequently occur with del(9q)s, as seen in this study (Table 1). When that happens, the prognostic significance of del(9q) seems to depend on the features of the additional alterations. Patients with del(9q) may have good prognosis when favorable genomic alterations coexist, as reported previously [Peniket et al., 2005; Balk et al., 2019]. The patients 1–4 in this study carried known favorable alterations, including t(15;17)(q24;q21), *PML*-*RARA*; t(8;21)(q22;q22.1), *RUNX1-RUNX1T1*, and biallelic pathogenic variants of *CEBPA.* Favorable prognoses were observed in these patients who stayed in complete remission until the last available follow-up (Fig. 1; Table 1). On the other hand, adverse prognoses may occur when del(9q)s coexist with genomic alterations of unknown or adverse diagnostic values. Reduced 5-year survival rates in AML with del(9q)and additional chromosome abnormalities have been reported [Peniket et al., 2005; Balk et al., 2019]. We further noticed that additional submicroscopic genomic alterations, including copy number alterations, copy number neutral loss of heterozygosities, and gene pathogenic variants, may also negatively affect the prognosis of AML with del(9q)s. The patients 5–7 and 9 in this study carried such additional alterations and showed a relatively short survival time (≤15 months) after standard AML chemotherapy regimens (Fig. 1; Table 1). The prognostic effects of the co-existing abnormalities are unknown. They might represent interactions between del(9q) and the co-existing genomic abnormalities or alteration of the treatment responses resulting from the co-existing abnormalities. This study also demonstrated the importance and advantage of using new technologies, such as SNP array and NGS, in cancer genomic analysis.

## Statement of Ethics

All patient-related data were collected retrospectively and anonymously. This study was conducted according to the guidelines in the Declaration of Helsinki; it was reviewed and approved by the Institutional Review Board of University of California San Francisco (IRB-10-01080).

## Conflict of Interest Statement

The authors have no conflicts of interest to declare.

## Funding Sources

The authors have no funding sources to declare.

## Author Contributions

Zhongxia Qi: study design, data analysis and interpretation, drafting of the manuscript. Kwun Wah Wen: study design, data analysis, critical revision of the manuscript for important intellectual content. Anita Ki: data acquisition and analysis. Sonam Prakash and Scott Kogan: study design, critical revision of the manuscript for important intellectual content. Jingwei Yu: study design, supervision, drafting and editing the manuscript.

## Data Availability Statement

The data that support the findings of this study are available from the corresponding author upon reasonable request.

## Figures and Tables

**Table 1:** Genomic findings, treatment and response of the AML patients with del(9q)s

Pt	Sex	Age, years	Treatment	OS, months	Karyotype	Copy number alterations (hg19)	Gene mutations
1	F	27	ATRA + As_2_O_3_	CR (>36)	47,XX,+8,del(9)(q13q22),t(15;17) (q24;q21)[19]/46,XX[1]	arr 8p23.3q24.3(164984_146293414)×3,9q13q22.33(68513625_10242699)×1[0.4],9q13q31.2 (6513625_11911316)×1[0.3], 15q26.1(91289405_91382995)×1	*FLT3* ITD

2	M	65	ATRA + As_2_O_3_	CR (>36)	46,XY,t(15;17)(q24;q21)[2]/47,idem,+8[5]/46,idem,del(9) (q13q34)[4]/46,XY[9]	arr(8)×3[0.1],9q13q31.3(70984372_112383429)x1[0.3]	

3	M	21	HiDAC	CR (>24)	47,XY,t(8;21)(q22;q22),del(9) (q12q31)[20]	arr 9q13q33.1(68513625_118744545)×1[0.5],10q25.3q26.11(118248041_120933771)×1[0.5]	

4	M	62	7+3	CR (>50)	46,XY,del(9)(q12q22)[5]/46,XY[15]	arr 9q21.11q22.32(70984372_97366400)×1[0.3]	*CEBPA* biallelic

5	F	35	7+3	12	46,XX,del(9)(q13q22)[20]	arr 9q21.11q31.2(70984372_109427621)×1[0.8],13q12.11q34(20358616_115103529)×2 hmz[0.6]	*FLT3* ITD

6	F	55	7+3	13	46,XX,?add(9)(q22)[20]	arr 6p22.3(17034315_18225493)×1[0.9],9q22.1q31.2(90590650_108808480)×1[0.9],9q31.3(112473382_114251696)×1[0.9],9q32(115460239_11715334)×1[0.9],9q34.11q34.13(132958305_134028856)×1[0.9], 13q12.12q34(23838241_115103529)×2 hmz[0.8]	*FLT3* ITD

7	F	51	7+3	15	46,XX,del(9)(q12q31)[5]/46,XX[15]	arr 9q21.11q31.1(70984372_106631091)×1[0.8]	*FLT3* ITD

8	F	73	7+3	22	45,X,–X,del(9)(q12q22)[20]	arr (X)×1[0.4],9q21.11q22.32(68838523_97888730)×1[0.3]	

9	M	37	7+3	12	46,XY,del(9)(q13q22)[16]/46,XY[14]	arr 6p25.3p22.1(165632_30242171)×2 hmz[0.5],9q21.11q31.1(70984372_105207670)×1[0.4]	*IKZF1* Q446*, *WT1* R380fs*72, splice site 872+1G>T, *TP53* G245C, *CEBPA* D107fs*52, *SMARCB1* R377H

Pt, patients; F, female; M, male; OS, overall survival; ATRA, all-trans retinoic acid; HiDAC, hi-dose cytarabine; 7+3, AML induction regimen using cytarabine/idarubicin; CR, complete remission.
